# The complete mitochondrial genome and phylogenetic analysis of the European map butterfly *Araschnia levana* (Insecta: Lepidoptera: Nymphalidae)

**DOI:** 10.1080/23802359.2020.1810163

**Published:** 2020-09-01

**Authors:** Mackenzie R. Alexiuk, Jeffrey M. Marcus, Melanie M. L. Lalonde

**Affiliations:** Department of Biological Sciences, University of Manitoba, Winnipeg, Canada

**Keywords:** Illumina sequencing, mitogenomics, Lepidoptera, Nymphalidae, Nymphalini

## Abstract

The European map butterfly *Araschnia levana* (Linnaeus, 1758) is a species showing extreme seasonal polyphenism. The complete 15,207 bp circular *A. levana* mitogenome consisting of 81.6% AT nucleotides, was assembled by Illumina genome skimming. It includes 22 tRNAs, 13 protein-coding genes, 2 rRNAs, and a control region in the typical butterfly gene order. *Araschnia levana COX1* features an atypical CGA start codon and *ATP6*, *COX1*, *COX2*, *ND1*, *ND3*, and *ND4* have incomplete stop codons completed by 3′A residues added to the mRNA. Phylogenetic reconstruction places *A. levana* as a basal lineage within tribe Nymphalini, consistent with previous phylogenetic hypotheses.

The European map butterfly, *Araschnia levana*, is a lepidopteran that displays seasonal polyphenism, having distinct long-day (LD) and short-day (SD) morphologies based on the length of daylight exposure in the last larval instar (Vilcinskas and Vogel 2016). The LD morphology prevalent in the summer displays brown and white coloration, contrasted with the SD morphology prevalent in the springtime after overwintering which displays red and black coloration (Windig and Lammar 1999; Nijhout 2010). Due to the differences in morphologies of wing coloration and other physical characteristics, *A. levana* was once classified as two different species by Linnaeus (who originally described the SD form as *Papilio levana* and the LD form as *P. prorsa* (Linnaeus 1758)) but later it was recognized that this was a single species displaying extreme seasonal polyphenism (Hübner 1816; Koch and Bückmann 1987). *Araschnia levana* is widely distributed throughout Europe, and has spread into extreme northern and southern regions due to the effects of climate change (Parmesan et al. 1999). Here, we report the complete mitochondrial genome sequence of *A. levana* (GenBank MT712075) from specimen AL2017.1, collected in Embourg, Belgium (GPS 50.590N, 5.607E) in April 2017, that has been pinned, spread, and deposited in the Wallis Roughley Museum of Entomology, University of Manitoba (voucher WRME0507734).

DNA was prepared (McCullagh and Marcus 2015) and sequenced by Illumina NovaSeq6000 (San Diego, CA) (Marcus 2018). The mitogenome of *A. levana* was assembled by Geneious 10.0.9 from 9,941,511 paired 150 bp reads using a *Mallika jacksoni* (Lepidoptera: Nymphalidae) reference mitogenome (MT704828) (Alexiuk et al. Submitted) and was annotated with respect to sequences from *M. jacksoni* and *Junonia stygia* (MN623383) (Living Prairie Mitogenomics Consortium 2020). The *A. levana* nuclear rRNA repeat (GenBank MT750296) was also assembled and annotated using the rRNA repeat from *M. jacksoni* (MT704831) as a reference sequence.

The *A. levana* circular 15,207 mitogenome assembly was composed of 5891 paired reads with nucleotide composition: 40.5% A, 10.9% C, 7.4% G, and 41.2% T. The gene composition and order in *A. levana* is identical to all known nymphalid mitogenomes (Linard et al. 2017). *Araschnia levana COX1* features an atypical CGA start codon as in many other insects (Liao et al. 2010). *Araschnia levana ND2* has an ATA start codon, which is used infrequently in insect mitochondria, but is fairly common in the mitochondria of some other animal groups (Okimoto et al. 1990; Han et al. 2016). The mitogenome contains two protein-coding genes (*COX1*, *COX2*) with single-nucleotide (T) stop codons, and four protein-coding genes (*ATP6*, *ND1*, *ND3*, *ND4*) with two-nucleotide (TA) stop codons completed by post-transcriptional addition of 3′A residues. The locations and structures of tRNAs were determined using ARWEN v.1.2 (Laslett and Canback 2008). tRNAs have typical cloverleaf secondary structures except for trnS (AGN) where the dihydrouridine arm is replaced by a loop, while the mitochondrial rRNAs and control region are typical for Lepidoptera (McCullagh and Marcus 2015).

We reconstructed a phylogeny using complete mitogenomes from *A. levana* and 41 additional mitogenomes from subfamily Nymphalinae (Lalonde and Marcus 2019a, 2019b; Chen et al. 2020; Alexiuk et al. Submitted; Hamilton et al. Submitted; Lalonde and Marcus Submitted; Payment et al. Submitted-a, Submitted-b). Mitogenome sequences were aligned in CLUSTAL Omega (Sievers et al. 2011) and analyzed by parsimony and maximum likelihood (model selected by jModeltest 2.1.7 (Darriba et al. 2012) and likelihood ratio test (Huelsenbeck and Rannala 1997) in PAUP* 4.0b8/4.0d78 (Swofford 2002) (Figure 1). Phylogenetic analysis places the *A. levana* mitogenome as the basal lineage among the available sequenced mitogenomes from the tribe Nymphalini, which is consistent with previous molecular phylogenetic studies of family Nymphalidae (Wahlberg et al. 2005, Wahlberg et al. 2009).

**Figure 1.**
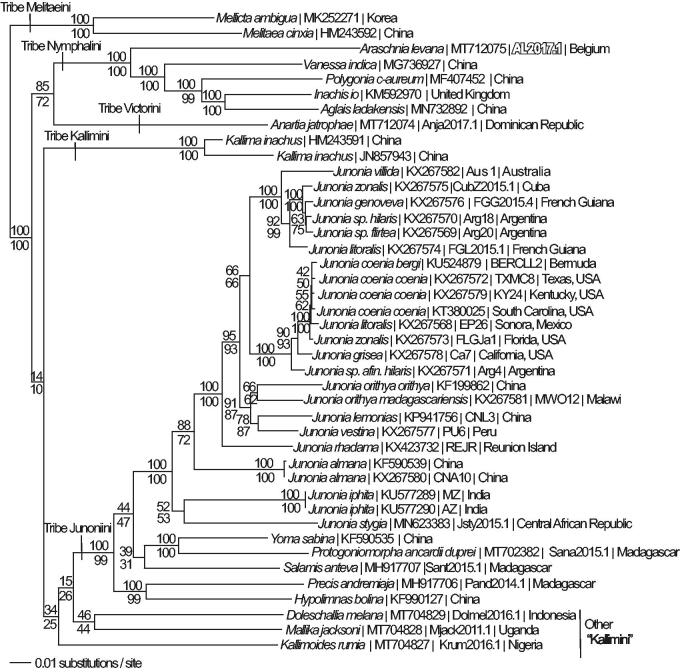
Maximum likelihood phylogeny (GTR + G model, *G* = 0.2330, likelihood score 117762.66543) of *Araschnia levana* (Tribe Nymphalini), 4 additional mitogenomes from tribe Nymphalini, 29 mitogenomes from tribe Junonini, 5 from Kallimini, 1 from Victorini, and 2 outgroup from tribe Melitaeini in subfamily Nymphalinae based on 1 million random addition heuristic search replicates (with tree bisection and reconnection). One million maximum parsimony heuristic search replicates produced 16 trees (parsimony score 20,698 steps) which differ from one another only by the arrangement of *Junonia coenia* mitogenomes and one of which has an identical tree topology to the maximum likelihood tree depicted here. Numbers above each node are maximum likelihood bootstrap values and numbers below each node are maximum parsimony bootstrap values (each from 1 million random fast addition search replicates).

## Data Availability

The data that support the findings of this study are openly available in GenBank of NCBI at https://www.ncbi.nlm.nih.gov, reference numbers MT712075 and MT750296.
